# Biallelic Truncating Variant in *LRGUK* Is Associated With Severe Multiple Morphological Abnormalities of the Sperm Flagella and Sperm Nuclear Defects in Humans

**DOI:** 10.1111/cge.70220

**Published:** 2026-07-23

**Authors:** Wiâme Mokkedem, Zeina Wehbe, Anne‐Laure Barbotin, Angèle Boursier, Célia Tebbakh, Nicolas Thierry‐Mieg, Marie Bidart, Antonella Migliaccio, Rosanna Chianese, Christophe Arnoult, Raoudha Zouari, Zine‐Eddine Kherraf, Pierre F. Ray, Guillaume Martinez, Charles Coutton

**Affiliations:** ^1^ Institute for Advanced Biosciences, Team Genetics Epigenetics and Therapies of Infertility University of Grenoble Alpes Grenoble France; ^2^ CHU Grenoble Alpes UM de Génétique Chromosomique Grenoble France; ^3^ CHU Lille Laboratoire de Biologie de la Reproduction‐Spermiologie‐CECOS Lille France; ^4^ Development and Plasticity of the Neuroendocrine Brain University of Lille Lille France; ^5^ CHU Grenoble Alpes UMGI‐DPI Grenoble France; ^6^ CNRS, University of Grenoble Alpes Grenoble France; ^7^ UM Génétique Moléculaire: Maladies Héréditaires et Oncologie University Hospital Grenoble Alpes Grenoble France; ^8^ Department of Experimental Medicine University of Campania L. Vanvitelli Naples Italy; ^9^ Polyclinique Les Jasmins Centre d’Aide Médicale à la Procréation Tunis Tunisia

**Keywords:** central apparatus, CFAP246, LRGUK, male infertility, MMAF, teratozoospermia

## Abstract

Multiple morphological abnormalities of the flagella (MMAF) is a severe form of male infertility characterized by immotile spermatozoa with absent, short, coiled, or irregular flagella. Despite advances in whole‐exome sequencing, many cases remain genetically unexplained. Here, we identify a homozygous truncating variant in *LRGUK* (c.1063C>T; p.Arg355Ter) in an infertile man presenting with a typical MMAF phenotype. The variant, identified by whole‐exome sequencing in a cohort of 168 MMAF patients and confirmed by Sanger sequencing, is predicted to result in loss of the C‐terminal guanylate kinase‐like domain. Immunofluorescence showed absence of LRGUK protein in patient spermatozoa, supporting a loss‐of‐function effect. Semen analysis revealed impaired motility and complete teratozoospermia. Morphological and ultrastructural analyzes demonstrated severe defects affecting both sperm head and flagellum, including disorganized axonemal architecture and central pair abnormalities. Nuclear analyzes showed increased nuclear size, defective chromatin compaction, and elevated DNA fragmentation. Immunostaining further indicated alterations of central apparatus‐associated proteins, supporting a role for LRGUK in C1b projection organization. These findings identify LRGUK as a novel gene involved in male infertility, essential for spermatid morphogenesis and flagellum assembly.

## Graphical abstract

A homozygous truncating variant in *LRGUK* results in loss of the LRGUK protein and causes multiple morphological abnormalities of the flagella (MMAF), disrupted axonemal architecture, central pair defects, abnormal chromatin organization, and severe male infertility, establishing *LRGUK* as a novel human infertility gene.

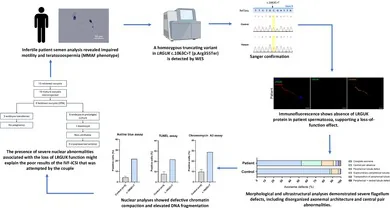

## 1 Introduction

Male infertility is recognized by the World Health Organization (WHO) as a major global health concern, affecting more than 50 million couples worldwide. Among infertile couples, male factors are involved in around 50% of cases, either alone or in combination with other factors [1]. Multiple morphological abnormalities of the flagella (MMAF) represent one of the most severe forms of male infertility. This phenotype is characterized by a heterogeneous association of absent, short, coiled, bent, or irregular flagella, typically associated with severe asthenozoospermia [2, 3, 4]. The advent of high‐throughput sequencing, notably whole‐exome sequencing (WES), has considerably improved the molecular diagnosis of male infertility [5]. WES has enabled the identification of over 40 genes implicated in MMAF [6], highlighting the marked genetic heterogeneity of this phenotype, which involves numerous genes encoding components of the axoneme and associated flagellar structures. Despite these advances, approximately 40%–50% of MMAF cases remain genetically unexplained [2, 7], indicating that additional genes essential for flagellum assembly remain to be identified. Clinically, establishing a precise genetic diagnosis is essential not only for etiological clarification and genetic counseling, but also because it significantly influences the management of MMAF patients, particularly in light of the expected outcomes of assisted reproductive technologies such as intracytoplasmic sperm injection (ICSI) [8].

*LRGUK* (Leucine‐Rich Repeats and Guanylate Kinase domain, formerly known as CFAP246) emerges as a compelling candidate gene in this context. The human LRGUK protein contains multiple leucine‐rich repeat (LRR) motifs, known mediators of protein–protein interactions [9], and a conserved guanylate kinase‐like (GUK) domain, typically implicated in cytoskeletal organization and multiprotein complex assembly [10]. Functional characterization in mice has shown that a truncating mutation in *Lrguk* results in complete male infertility associated with severe sperm head abnormalities and absent or malformed flagella [11]. In murine models, LRGUK localizes dynamically along the acrosome–acroplaxome–manchette–tail axis during spermiogenesis, implicating it in both nuclear shaping and flagellar assembly. Its loss leads to disorganized manchette microtubules, defective basal body docking, and failure of axoneme extension [12]. Despite strong evidence in mice, the role of LRGUK in human male infertility has not yet been studied. Whether LRGUK deficiency in humans results solely in flagellar defects or also disrupts nuclear morphogenesis and chromatin integrity remains unknown.

Here, we report for the first time a homozygous truncating variant in *LRGUK* in an infertile patient presenting with an MMAF phenotype. We aimed to characterize the molecular consequences of this loss‐of‐function variant, describe the associated flagellar and nuclear defects in spermatozoa, and investigate the impact on known LRGUK partners and architecture components of the flagella. By integrating genetic and cellular analyzes, this study seeks to establish *LRGUK* as a novel human infertility gene and to provide insight into the coordinated mechanisms linking nuclear shaping and flagellum biogenesis during spermiogenesis.

## 2 Materials and Methods

### 2.1 Patient Inclusion and Ethical Approval

The patient included in this study was recruited at the Clinique des Jasmins (Tunis, Tunisia) during routine clinical evaluation for infertility. Written informed consent was obtained from the participant in accordance with local regulatory requirements and the ethical principles of the Declaration of Helsinki. The study protocol was reviewed and approved by the relevant institutional ethics committees.

Biological samples were stored either at the CRB Germethèque (certified ISO‐9001 and NF‐S 96‐900) according to a standardized procedure or were part of the Fertithèque collection declared to the French Ministry of health (DC‐2015‐2580) and the French Data Protection Authority (DR‐2016‐392).

### 2.2 Semen Analysis

Semen samples were obtained by masturbation after 3 days of sexual abstinence. Following collection, samples were incubated at 37°C for 30 min to allow complete liquefaction. Semen parameters were assessed according to the World Health Organization (WHO) 5th edition guidelines (2010). The following parameters were recorded: ejaculate volume, pH, viscosity, sperm concentration, vitality, motility, and morphology. Morphological evaluation was performed on stained smears, and at least 200 spermatozoa were examined per slide.

### 2.3 Whole Exome Sequencing

The patient was included in a larger genomic screening cohort comprising 168 MMAF‐affected individuals. Briefly, coding regions along with intron/exon boundaries were enriched using the Exon V6 kit from Agilent Technologies (Wokingham, UK), followed by sequencing on an Illumina HiSeq X platform by a contracted service provider (Novogene, Cambridge, UK). Exome data were analyzed using an in‐house developed bioinformatics pipeline, comprising two modules available on GitHub under the GNU General Public License v3.0: https://github.com/ntm/grexome‐TIMC‐Primary and https://github.com/ntm/grexome‐TIMC‐Secondary. The analysis was conducted in two complementary steps. First, all variants (regardless of their predicted functional impact) were systematically screened against a curated and regularly updated panel of genes known to be associated with MMAF and male infertility, with filtering based on minor allele frequency (MAF < 1% in gnomAD v2.0 and < 3% in the 1000 Genomes Project phase 3) and compatible inheritance models. Second, an unbiased genome‐wide analysis was performed across all variant classes, filtered solely on the basis of MAF thresholds, to detect potential pathogenic variants in genes not previously associated with the phenotype. No additional pathogenic variant in a known MMAF or male infertility gene was identified, and the homozygous nonsense variant in LRGUK (c.1063C>T; p.Arg355Ter) remained the sole candidate meeting our prioritization criteria.

### 2.4 Sanger Sequencing

The newly identified *LRGUK* variant underwent validation through Sanger sequencing conducted on an ABI 3500XL instrument (Applied Biosystems) and data analysis was conducted using SeqScape software (AppliedBiosystems).

### 2.5 Transmission Electron Microscopy

Transmission electron microscopy (TEM) experiments were performed like previously [13]. using sperm cells from fertile control and patient P0582. After fixation in 2.0% v/v glutaraldehyde in phosphate buffer (pH 7.4), the sperm pellet underwent a 15 min wash in fresh buffer containing 4% w/v sucrose and was subsequently embedded in 2% agar. Postfixation involved the use of 1% osmic acid in phosphate buffer. Following fixation, small pieces of agar containing spermatozoa were dehydrated through a graded series of ethanol. Subsequently, these pieces were further embedded in Epon resin (Polysciences Inc., Warrington, Pennsylvania, USA). Sections were then cut using a Reichert OmU2 ultramicrotome (Reichert‐Jung AG, Vienna, Austria) equipped with a diamond knife. Ultrathin sections (70 nm) were collected on Parlodion 0.8%/isoamyl acetate‐coated 100 mesh Nickel grids (EMS, Fort Washington, Pennsylvania, USA) and counterstained with 2% uranyl acetate and lead citrate before observation. Examination of the sections was conducted using a Zeiss transmission electron microscope 902 (Leo, Rueil‐Malmaison, France), and images were acquired utilizing a Gatan Orius SC1000 CCD camera (Gatan France, Grandchamp, France).

### 2.6 Immunofluorescence Analysis

Immunofluorescence (IF) was performed to assess the expression and localization of LRGUK and selected flagellar proteins. Ejaculated spermatozoa were fixed in 4% paraformaldehyde and stored at −20°C until use. Samples were then permeabilized and blocked in PBS containing 0.1% Triton X‐100 and 2% normal goat serum. Slides were incubated overnight at 4°C in a humidified, light‐protected chamber with the following primary antibodies: Anti‐LRGUK (rabbit polyclonal, Novus Biologicals, 1:100); Anti‐*α*‐tubulin (mouse monoclonal, 1:200). The anti‐LRGUK antibody was further validated by Western blotting, which reproduced the banding pattern consistently reported by the supplier (Figure S1). Additional antibodies targeting candidate LRGUK partners and axonemal markers included CFAP70, SPEF2, SPAG6 (central pair complex); GAS8 (nexin‐dynein regulatory complex); RSPH4A (radial spokes); DNALI1 (inner dynein arms); IFT20 (intraflagellar transport); as well as HOOK1, HOOK2, and MYCBPAP (predicted LRGUK partners). Fluorophore‐conjugated secondary antibodies (Alexa Fluor 488 goat anti‐rabbit and Alexa Fluor 568 goat anti‐mouse) were applied following washing steps. Nuclei were counterstained with DAPI. Slides were mounted using Dako Fluorescent Mounting Medium and sealed. Images were acquired using a fluorescence microscope equipped with DAPI, FITC, and Rhodamine filters. Acquisition parameters were kept identical for patient and control samples. Image processing was performed using Ikaros software (Metasystems).

### 2.7 Nuclear Morphology Analysis

Quantitative nuclear morphology assessment was performed using Nuclear Morphology Analysis Software (version 2.1). High‐resolution images of DAPI‐stained nuclei were analyzed to quantify shape parameters, including area, perimeter, elongation, and symmetry indices.

### 2.8 TUNEL Assay

DNA fragmentation was assessed using the TUNEL assay (Roche In Situ Cell Death Detection Kit, Fluorescein). Frozen semen samples were thawed, washed in PBS, and fixed in methanol/acetic acid (3:1). Smears were prepared on Superfrost slides and stored at −20°C until use. After permeabilization with 0.1% Triton X‐100 in sodium citrate, slides were incubated with labeling mix for 1 h at 37°C in the dark. Negative controls were processed without terminal transferase enzyme. Slides were washed, counterstained with DAPI, and mounted. At least 200 spermatozoa per slide were analyzed at 100× magnification. The DNA fragmentation index (DFI) was calculated as the percentage of TUNEL‐positive spermatozoa.

### 2.9 Aniline Blue Staining

Aniline blue coloration was performed on ejaculated spermatozoa from the patient and fertile control. After washing twice with 5 mL of PBS 1×, a small portion of semen samples was fixed with a 3% glutaraldehyde solution in PBS 1× for 30 min at room temperature. Slides were then incubated in a succession of baths: 5 min in water, 10 min in 5% aniline blue diluted in 4% acetic acid solution, twice for 2 min in water, 2 min in 70%, 90%, and 100% ethanol solutions and finally for 2 min in toluene. Slides were then analyzed using a transmitted light microscope at 100× objective with oil. At least 200 spermatozoa per slide were analyzed with dark blue cells considered as positive, and lightly to very lightly stained cells considered as negative.

### 2.10 Chromomycin A3 (CMA3) Staining

CMA3 staining was performed to assess protamine deficiency. Slides were equilibrated in McIlvaine buffer (pH 7.0, containing 10 mM MgCl_2_), then incubated for 20 min with CMA3 (25 mg/L). Following washing, slides were air‐dried, counterstained with DAPI, and mounted. Fluorescence microscopy was performed using appropriate filters, with CMA3 fluorescence visualized in the green channel. Increased CMA3 fluorescence was interpreted as indicative of impaired chromatin protamination, with at least 200 spermatozoa counted per slide.

## 3 Results

### 3.1 Whole‐Exome Sequencing Reveals a Homozygous Loss‐Of‐Function Variant in *LRGUK*

Whole‐exome sequencing (WES) was performed in a cohort of 168 infertile men presenting with multiple morphological abnormalities of the flagellum (MMAF). Variant filtering identified a homozygous nonsense variant in *LRGUK* (NM_144648.3: c.1063C>T; p.Arg355Ter) in one patient of Algerian origin (Tebessa, Algeria) (Figure 1A). The variant is present in the Genome Aggregation Database (gnomAD v4.1.1, http://gnomad.broadinstitute.org/) database with a minor allele frequency (MAF) of 3.75e‐6. The patient reported a familial history of infertility, including three infertile uncles and one infertile brother, suggesting a possible autosomal recessive inheritance pattern consistent with the homozygous state of the variant. Unfortunately, pedigree analysis could not be performed to confirm the transmission of the variant as the samples of his relatives were not available. The variant was subsequently validated by Sanger sequencing (Figure 1B). The variation introduces a premature termination codon, predicted to truncate the protein at residue 355 and remove the C‐terminal guanylate kinase–like (GUK) domain (Figure 1C,D). The arginine residue at position 355 is conserved across mammalian species (Figure S2), further supporting the functional relevance of this region.

**FIGURE 1:**
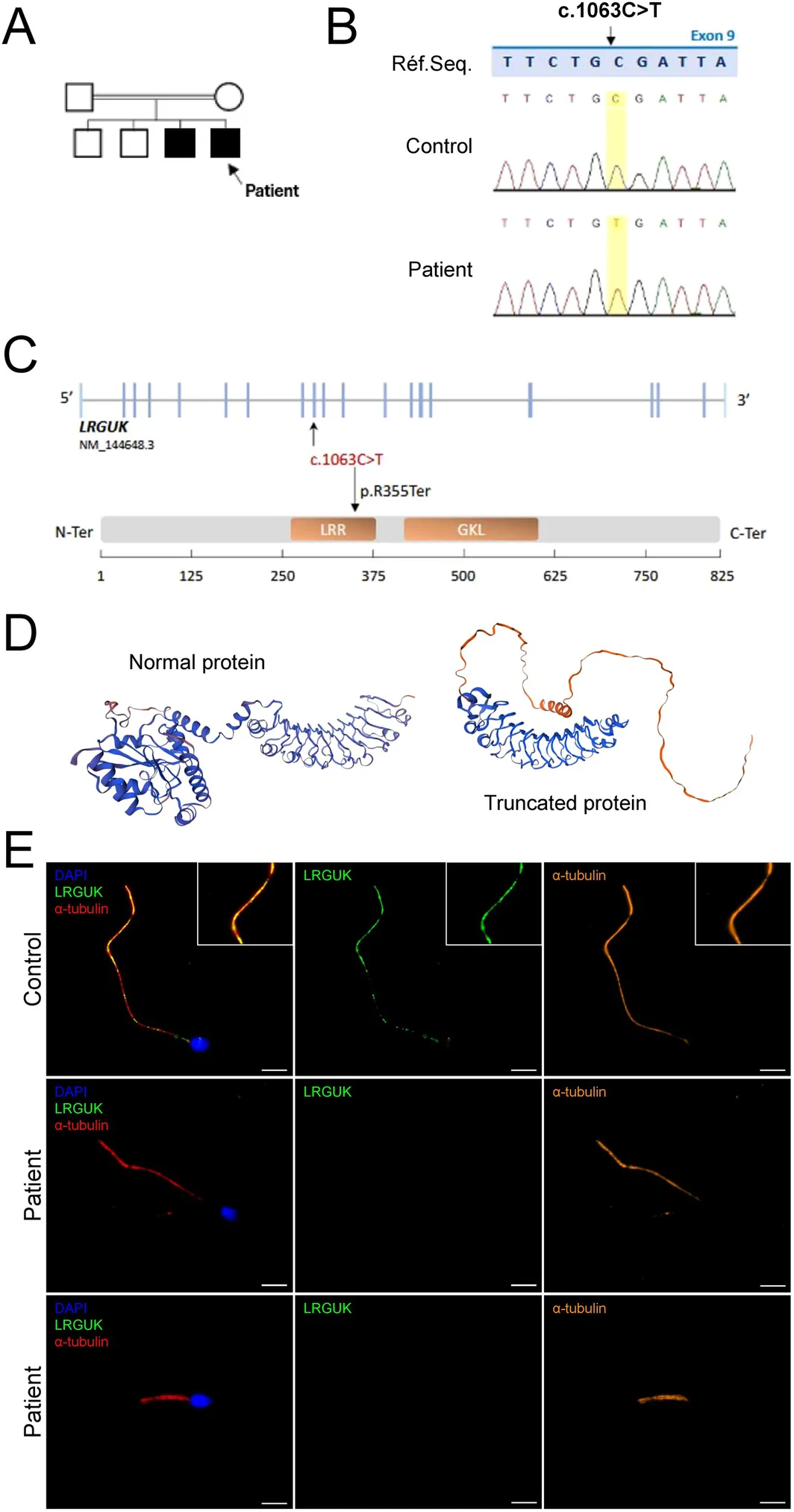
(A) Pedigree analysis. Both the proband (arrow) and his brother displayed an infertility phenotype. (B) Electropherogram from Sanger sequencing indicating the presence of c.1063C>T (NM_144648.3). (C) Position of the observed variant on the structure of the canonical transcript of *LRGUK*. (D) Impact on the LRGUK protein is visually represented using modeling from SWISS‐MODEL for the truncated protein. (E) LRGUK immunostaining of sperm cells obtained from a fertile control individual and our patient. Sperm cells were subjected to labeling using an anti‐LRGUK antibody (green), an anti‐acetylated tubulin antibody (red), and DAPI (blue) for nuclear staining. In the fertile control, the LRGUK signal was detected with high intensity throughout the entire length of the flagellum. Conversely, in all sperm from the patient, regardless of their morphology, the LRGUK signal was completely absent. No antibody signals were detected in the negative control. Scale bars: 10 μm. [Colour figure can be viewed at wileyonlinelibrary.com]

To further investigate the pathogenicity of the *LRGUK* variant identified, we examined the distribution of LRGUK in sperm cells from fertile controls and the patient by immunofluorescence staining. In control spermatozoa, LRGUK displayed a punctate staining pattern along the flagellum, consistent with its previously described localization along the axoneme [11]. In contrast, no detectable LRGUK signal was observed in spermatozoa from the patient, while α‐tubulin staining remained preserved, indicating that axonemal microtubules were still detectable (Figure 1E). These findings confirm the deleterious effect of the identified c.1063C>T variation in the *LRGUK* gene.

### 3.2 *LRGUK* Variant Leads to Severe Structural and Ultrastructural Sperm Defects

Semen analysis revealed markedly abnormal sperm (Table 1). Both semen samples were collected by masturbation, processed within 1 h of ejaculation, and analyzed according to WHO 2010 guidelines. The patient's clinical condition at the time of each collection was not recorded, as these assessments were performed as part of routine clinical care. The initial spermogram showed a normal semen volume (2.5 mL) and sperm concentration (47 × 10^6^/mL). However, total motility was reduced to 30%, with only 5% progressive motility, values below the WHO 2010 reference thresholds (≥ 40% total motility and ≥ 32% progressive motility). A second spermogram performed prior to an IVF‐ICSI procedure showed a further deterioration of motility parameters, with only 5% total motility and complete absence of progressive motility (0%). Such inter‐sample variability in motility is a well‐recognized physiological phenomenon and does not affect the morphological assessment. Morphological evaluation of spermatozoa confirmed complete teratozoospermia, with 100% abnormal morphologies. Multiple structural defects were observed affecting the sperm head, midpiece, and flagellum. Head abnormalities were highly prevalent, including amorphous acrosomes (94%), abnormal head base (60%), elongated heads (24%), microcephalic heads (10%), and round heads (2%). Midpiece defects were also frequent and consisted of cytoplasmic residues (28%), angulated midpieces (20%), and abnormally thick midpieces (10%). Flagellar abnormalities were particularly prominent, including irregular flagellar caliber (50%), coiled flagella (18%), absent flagella (16%), and short flagella (8%). The combination of multiple flagellar malformations together with the impaired motility is consistent with the diagnostic criteria of the MMAF phenotype.

**TABLE 1:** Detailed semen parameters of the patient.

Date	10/06/2023	14/06/2023	Normal range
Specimen characteristics			
Abstinence duration (days)	3 days	4 days	2–7
Volume (mL)	2.5	2	> 1.5
Viscosity	Normal	na	
Numeration			
Sperm count (10^6^/mL)	47	39	> 15
Round cells (10^6^/mL)	5	na	< 5
Polynuclear (10^6^/mL)	0	na	< 1
Motility			
Progressive sperm (%)	5	0	> 32
Nonprogressive sperm (%)	30	5	*P* + NP > 40
Immotile sperm (%)	70	95	< 60
Other tests			
Vitality (% alive)	68	na	> 58
Morphology			
Normal (%)	0	na	> 4
Abnormal (%)	100	na	< 96
Head anomaly (%)			
*Elongated (%)*	24	na	
*Thinned (%)*	6	na	
*Microcephalic/Pinhead (%)*	10	na	
*Globocephalic/Round (%)*	2	na	
*Macrocephalic/Multiple (%)*	2	na	
*Abnormal base (%)*	60	na	
*Malformed acrosome (%)*	94	na	
Intermediate piece anomaly (%)			
*Cytoplasmic residue (%)*	28	na	
*Thinned (%)*	0	na	
*Thickened (%)*	10	na	
*Angulation (%)*	20	na	
Flagellum anomaly (%)			
*Absent (%)*	16	na	
*Short (%)*	8	na	
*Irregular size (%)*	50	na	
*Coiled (%)*	18	na	
*Multiple (%)*	0	na	

To determine the origin of the defects observed in the semen analysis, a detailed morphological examination of spermatozoa was performed using optical microscopy following Harris–Schorr staining. This analysis revealed a broad spectrum of structural abnormalities affecting both the sperm head and flagellum (Figure 2A). At the head level, spermatozoa frequently exhibited asymmetrical, rounded, and tapered heads. Many nuclei appeared poorly elongated and irregularly shaped, suggesting defects in the cytoskeletal remodeling processes responsible for head shaping during spermiogenesis. The flagellum was also profoundly altered. Numerous spermatozoa lacked a visible flagellum, whereas others displayed markedly shortened tails, indicative of defective axoneme assembly or impaired flagellar elongation. In addition, several spermatozoa exhibited coiled flagella forming tight loops, often wrapped around the sperm head or midpiece, a configuration typically associated with structural disorganization of axonemal or peri‐axonemal components.

**FIGURE 2:**
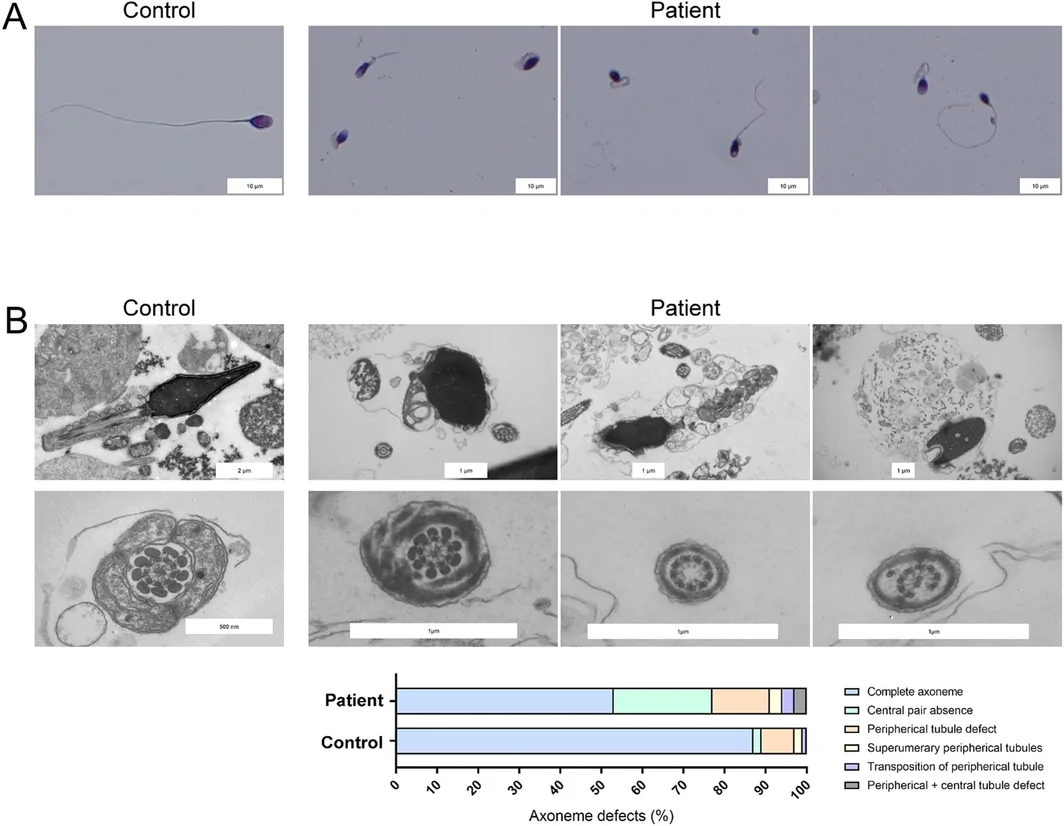
(A) Light microscopy analysis of spermatozoa from a fertile control individual and the patient with the *LRGUK* variant. Patient spermatozoa displayed a broad spectrum of morphological abnormalities affecting both the head and flagellum, including asymmetrical, rounded and tapered heads with irregular nuclear shape, as well as severe flagellar defects such as absent or markedly shortened tails and coiled flagella frequently wrapped around the head or midpiece. Scale bar = 10 μm. (B) Transmission electron microscopy analysis of spermatozoa from a fertile control individual and the patient. Control spermatozoa displayed normal flagellar ultrastructure. In contrast, patient spermatozoa exhibited severe ultrastructural defects, including central pair absence (25%), peripheral tubule defects (13%), supernumerary peripheral tubules (3%), transposition of peripheral tubule (3%) and peripheral and central tubule defects (3%). Longitudinal sections also revealed truncated flagella, disorganization of the mitochondrial and fibrous sheath, as well as cytoplasmic inclusions containing unassembled axonemal components, indicative of defective flagellum assembly. [Colour figure can be viewed at wileyonlinelibrary.com]

Next, transmission electron microscopy (TEM) was performed to analyze the ultrastructure of the sperm flagellum. In control spermatozoa, longitudinal and cross‐sectional views revealed the typical organization of the flagellum, including the axoneme, outer dense fibers (ODFs), mitochondrial sheath of the midpiece, and fibrous sheath of the principal piece (Figure 2B). In contrast, in the patient spermatozoa, the mitochondrial sheath appeared disorganized in the midpiece, and the axonemal architecture was severely disrupted (53% of complete axoneme vs. 90% in the control), with absence of the central pair complex (25% of axonemal defects), frequent absence of radial spokes, and irregular arrangement of the outer microtubule doublets (22% of axonemal defects). In addition, fibrous sheath disorganization was observed in several sections. Longitudinal sections further revealed severely truncated flagella and the presence of cytoplasmic structures containing unassembled axonemal components, indicating defective flagellum assembly.

Together, these findings demonstrate structural abnormalities affecting both head morphology and flagellar ultrastructural organization, providing a structural basis for the infertility phenotype observed in the semen analysis.

### 3.3 *LRGUK* Loss‐Of‐Function Is Associated With Defects of the Central Pair

We next investigated the flagellar and axonemal defects of the *LRGUK* variation. Immunostaining of DNALI1, GAS8, RSPH1, and AKAP4 was comparable with that observed in control cells, suggesting that inner dynein arms (IDAs), outer dynein arms (ODAs), Nexin‐Dynein regulatory complex (NDRC), the radial spokes (RS), and the fibrous sheath, respectively, were not directly affected by variations in *LRGUK* (Figure S3A).

In the same way, immunostaining of LRGUK's predicted partners [14, 15], namely CFAP70, MYCBPAP, HOOK1, and HOOK2 did not show any difference in staining in the patient's sperm compared to the control cells (Figure S3B), suggesting that variations in *LRGUK* do not directly impact the stability of these complexes in human sperm cells. Immunostaining of IFT protein IFT20 (Figure S3C), for which a role of LRGUK has been suggested in mouse models [11], did not seem to reveal any alteration in the patient's sperm.

Next, given that LRGUK has been predicted by computational studies to localize to the C1b projection of the central apparatus (CA), we examined the localization of SPEF2, CFAP69, and SPAG6, which are known components of the C1 projection [15]. Interestingly, SPEF2 and SPAG6 appeared reduced in intensity and showed a more fragmented or disorganized distribution compared to the control (Figure 3A). Notably, staining for CFAP69 showed a notable absence of signal in the patient's spermatozoa (Figure 3B). *LRGUK* loss‐of‐function thus appears to lead to a severe defect of the CA, which is consistent with its localization in the C1b projection [15].

**FIGURE 3:**
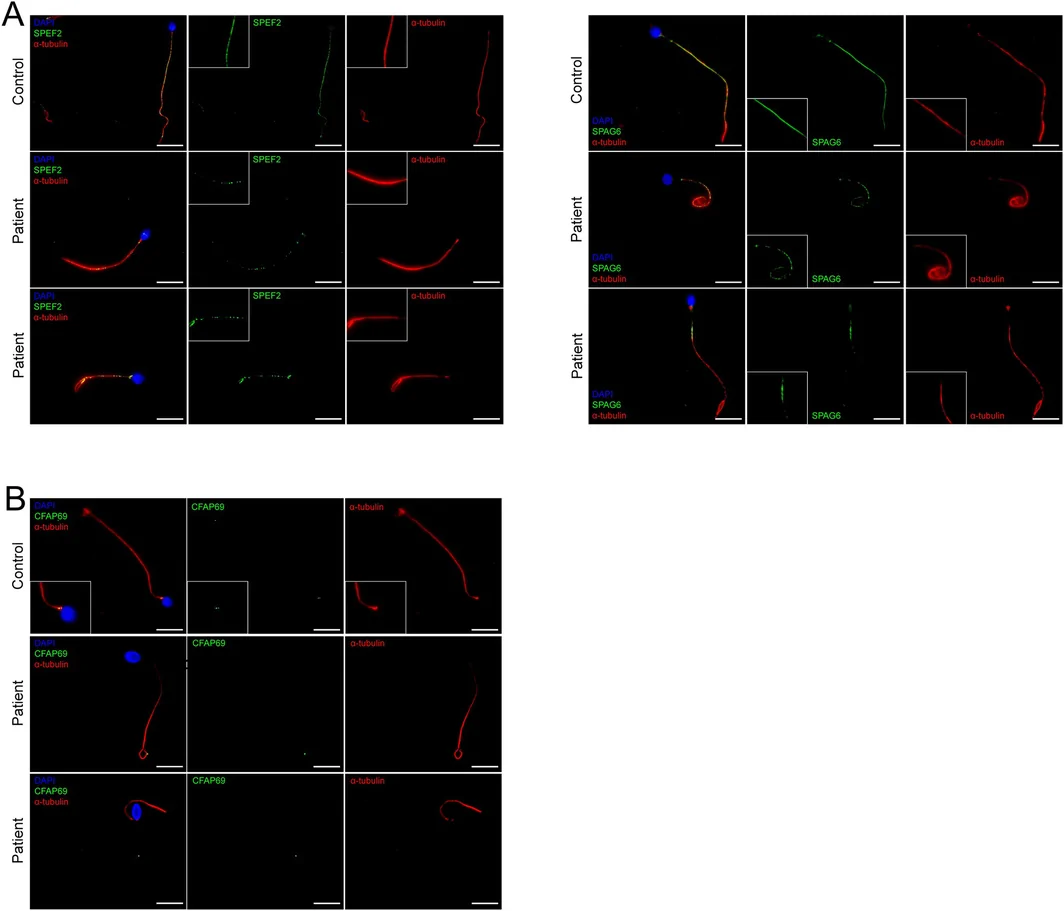
Immunostaining of central apparatus proteins in spermatozoa from the LRGUK patient. Sperm cells from a fertile control individual and the patient were stained with antibodies against SPEF2, SPAG6, and CFAP69 (green), together with anti‐acetylated tubulin (red). DNA was counterstained with DAPI (blue). In control spermatozoa, all markers showed a continuous distribution along the flagellum. In contrast, in the patient, SPEF2 and SPAG6 displayed reduced intensity and a fragmented, disorganized pattern (A). CFAP69 staining was absent (B). No antibody signals were detected in the negative control. Scale bars: 10 μm. [Colour figure can be viewed at wileyonlinelibrary.com]

### 3.4 *LRGUK* Variant Is Associated to Nuclear Abnormalities and Chromatin Integrity Defects

We next sought whether the structural abnormalities observed in sperm head morphology were associated with defects in nuclear organization; quantitative analysis of DAPI‐stained sperm nuclei was performed. Image‐based morphometric measurements revealed a significant increase in nuclear size in the patient's spermatozoa compared with control cells, but also a broader dispersion of values, indicating increased heterogeneity in nuclear dimensions (Figure 4A).

**FIGURE 4:**
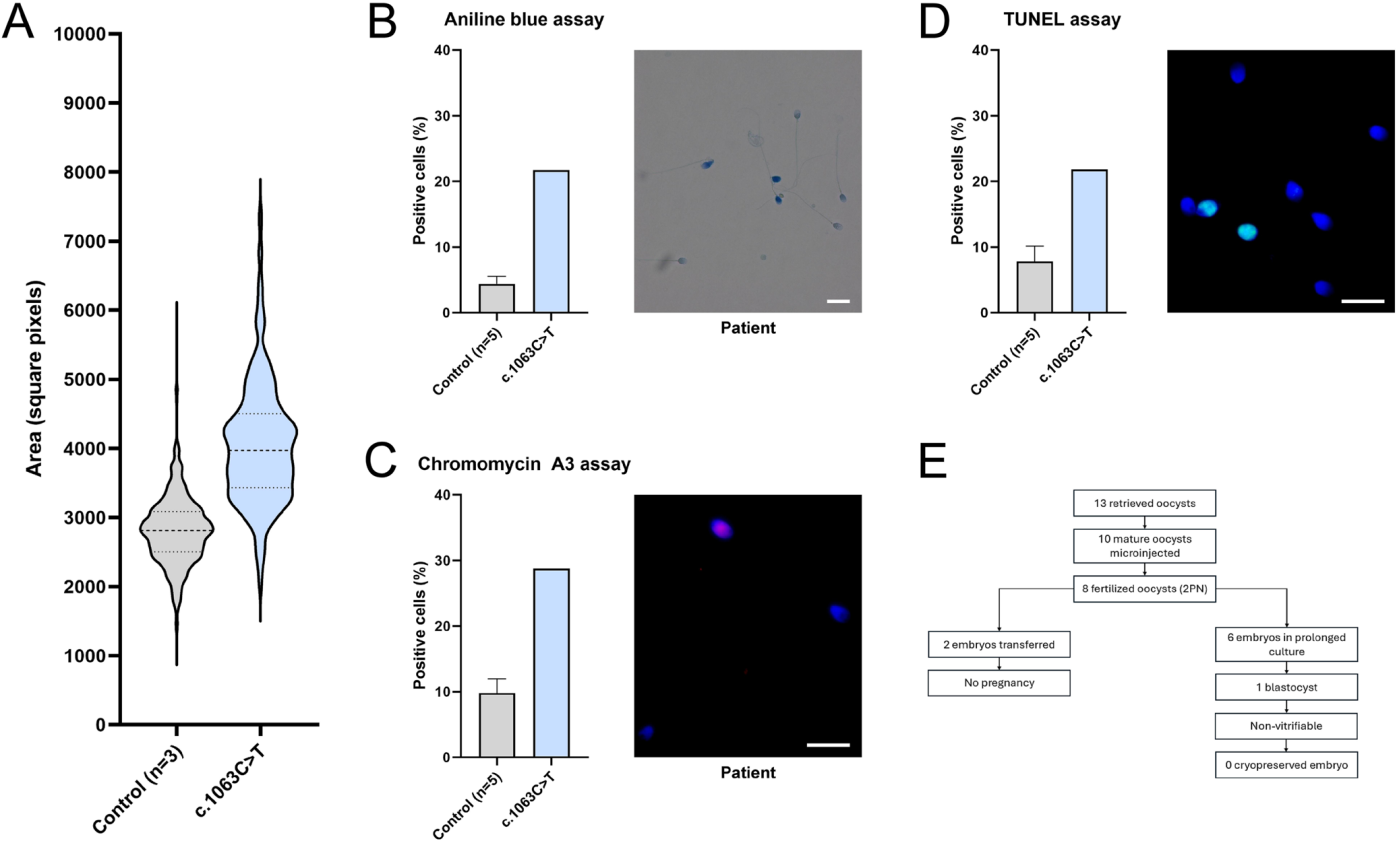
(A) Quantitative analysis of sperm nuclear size. Violin plots representing the distribution of nuclear area (square pixels) in DAPI‐stained spermatozoa from three fertile controls and the patient. The patient exhibits a significant increase in nuclear size compared to the control, along with a broader distribution, indicating increased heterogeneity in nuclear dimensions. (B) Aniline blue staining of sperm chromatin revealed an increased proportion of dark blue‐stained spermatozoa in the patient (22%) compared to the control (*n* = 5), indicating abnormal histone retention and defective chromatin compaction. (C) CMA3 staining showed a higher percentage of positive spermatozoa in the patient (29%) compared to the control (*n* = 5), consistent with impaired protamination and increased chromatin immaturity. (D) TUNEL assay showed elevated DNA fragmentation in the patient sample (22%) compared to the control (*n* = 5), reflecting reduced genomic integrity. Histograms display the results for each experiment (left), and representative images are shown (right). (E) Outcome of IVF–ICSI procedure in the couple. Two developing embryo were transferred without live birth. Of the remaining embryos, only one reached the blastocyst stage but was nonvitrifiable and could not be cryopreserved. Scale bars: 10 μm. [Colour figure can be viewed at wileyonlinelibrary.com]

In the light of this result, we hypothesized that this condition might be associated with DNA compaction defects. Subsequently, we conducted blue aniline staining, which uses the capacity of this acidic dye to bind to lysine residues to discriminate between lysine‐rich histones and arginine‐ and cysteine‐rich protamines [16]. The patient sample exhibited a marked increase in aniline blue positivity, with 22% of spermatozoa showing positive dark blue staining, indicating abnormal histone presence (Figure 4B). Chromomycin A3 (CMA3) staining was also performed. CMA3 is a fluorescent dye that binds preferentially to GC‐rich regions of DNA, with accessibility inversely correlated with protamine incorporation, indicating defective protamination and immature chromatin structure [17]. Quantitative analysis revealed that 29% of spermatozoa from the patient were CMA3‐positive, demonstrating a significant increase in chromatin immaturity and confirming the presence of defective chromatin packaging (Figure 4C). Next, we examined whether the chromatin abnormalities observed were associated with compromised DNA integrity using a TUNEL assay [18]. The results showed elevated DNA fragmentation in 22% of the patient's sperm (Figure 4D), indicating reduced genomic integrity.

Taken together, these results support the presence of severe nuclear abnormalities associated with the loss of *LRGUK* function and can partly explain the poor results of the IVF‐ICSI that was attempted by the couple (Figure 4E). During that attempt, 13 oocytes were collected, 10 were injected, resulting in eight fertilized zygotes, all of which developed into embryos. This led to two embryo transfers, but no live birth occurred. Of the other six embryos, only one developed to a blastocyst that was nonvitrifiable and thus couldn't be cryopreserved.

## 4 Discussion

In this study, we report the identification of a homozygous truncating variant in *LRGUK* in a patient presenting with severe male infertility characterized by a MMAF phenotype. Functional analyzes revealed an absence of the LRGUK protein in patient spermatozoa, together with structural abnormalities affecting both the axonemal architecture and sperm nuclear integrity. While the study is limited by the analysis of a single patient, the concordance between the genetic findings, the absence of protein expression, and the severe flagellar phenotype strongly supports a causal relationship between the *LRGUK* loss‐of‐function variant and the observed infertility phenotype. Although immunofluorescence confirmed the absence of LRGUK protein in patient spermatozoa, whether the identified nonsense variant triggers nonsense‐mediated mRNA decay (NMD) or leads to the production of a truncated, unstable protein could not be determined due to the unavailability of more biological material. Formal re‐evaluation of the variant according to ACMG/AMP guidelines supports a classification of Likely Pathogenic (Class 4), based on PVS1 (moderate), PS3 (moderate), PM2 (supporting), and PP4 (supporting) criteria.

Morphological and ultrastructural analyzes suggest that LRGUK plays an important role in maintaining the structural integrity of the axoneme. In particular, the ultrastructural alterations observed are compatible with defects affecting the projections of the central pair apparatus, which are essential for proper axonemal organization and motility regulation [19, 20]. Previous studies in murine models have proposed that LRGUK is involved in intra‐manchette transport (IMT), a microtubule‐based trafficking pathway responsible for delivering structural proteins during spermiogenesis [11]. In mice, loss of *Lrguk* leads to severe abnormalities of both the sperm head and flagellum, associated with defects in manchette organization and basal body docking. In our study, we did not observe such defects, and our analyzes did not reveal clear alterations of proteins involved in IMT. However, these observations must be interpreted with caution as our analyzes were performed exclusively on mature ejaculated spermatozoa, and further investigations will be necessary to fully assess the potential involvement of *LRGUK* during earlier stages of spermiogenesis. Since IMT is active primarily during spermatid development, defects occurring at earlier stages of spermiogenesis cannot be directly assessed. The absence of testicular samples or immature germ cells in our study limits our ability to evaluate the potential involvement of LRGUK in IMT during human spermiogenesis.

Recent structural studies have determined LRGUK to be part of the C1b projection of the CA [15]. The CA consists of two singlet microtubules surrounded by multiple protein projections that interact with radial spokes to regulate dynein activity and coordinated flagellar beating. Disruption of these projections can therefore alter axonemal architecture and motility. Our results suggest that LRGUK could have a role in stabilizing the C1b projection in the human sperm flagellum. In this context, LRGUK may function as a structural component of the central pair complex. Furthermore, variations in several genes encoding components of the central pair apparatus have been reported to cause either isolated male infertility [21, 22] or combined phenotypes involving both infertility and primary ciliary dyskinesia (PCD) [23]. Whether these differences reflect variations in protein composition between cilia and flagella or cell‐type specific functional requirements remains unclear. Comparative structural studies, including cryo‐electron microscopy analyzes of central pair complexes in different cell types, will be necessary to better understand these potential differences.

In addition to flagellar defects, our analyzes revealed alterations in sperm nuclear morphology and chromatin organization in the patient, suggesting that LRGUK deficiency may also affect nuclear remodeling during spermiogenesis. Although the mechanisms underlying these nuclear abnormalities remain unclear, they may reflect indirect consequences of cytoskeletal defects occurring during sperm differentiation.

From a clinical perspective, the identification of *LRGUK* variants as a cause of male infertility has important implications for genetic diagnosis and patient management. Despite the severe flagellar defects observed in MMAF patients, fertilization may still be achievable through assisted reproductive technologies such as intracytoplasmic sperm injection (ICSI). However, establishing a precise molecular diagnosis appears essential for appropriate genetic counseling and for assessing potential risks of transmission to offspring. Further studies involving additional patients will be necessary to better define the spectrum of phenotypes associated with LRGUK's loss‐of‐function.

Taken together, our findings support a critical role for LRGUK in the structural organization of the human sperm flagellum and provide evidence that loss‐of‐function variants in this gene can lead to severe male infertility associated with the MMAF phenotype. Future investigations combining genetic analyzes, high‐resolution structural approaches, and functional studies in germ cell models will be required to fully elucidate the molecular mechanisms through which LRGUK contributes to spermatid morphogenesis and flagellar assembly in humans.

## Funding

This work was supported by the Université Grenoble Alpes, the Institut National de la Santé et de la Recherche Médicale, the Conseil National de la Recherche Scientifique, and the Agence Nationale de la Recherche (ANR‐21‐CE17‐0007, ANR‐19‐CE17‐0014).

## Conflicts of Interest

The authors declare no conflicts of interest.

## Supporting information


**Figure S1:** Validation of the anti‐LRGUK antibody by Western blotting. (Left) Supplier‐provided immunoblot of control and LRGUK‐overexpressing cell lysates. (Right) In‐house immunoblot reproducing the reported banding pattern. A prominent band is detected near the predicted molecular mass of LRGUK, along with an additional band at approximately 50–55 kDa. Arrows indicate corresponding bands. Molecular masses are in kilodaltons (kDa).

**Figure S2:** Conservation of the LRGUK protein sequence around residue Arg355 across mammalian species. Multiple sequence alignment of the LRGUK protein region encompassing residue Arg355 across 18 mammalian species. The arginine residue at position 355 (R) is conserved across all species examined. Conservation scores are indicated below the alignment.

**Figure S3:** Immunostaining of axonemal structures, LRGUK partners, and IFT protein in spermatozoa from the LRGUK patient. Sperm cells from a fertile control individual and the patient were stained with antibodies against DNALI1, GAS8, RSPH1, and AKAP4 (green), together with anti‐acetylated tubulin (red). DNA was counterstained with DAPI (blue). (A) Comparable staining patterns were observed between control and patient spermatozoa, indicating no obvious defects in inner dynein arms (DNALI1), nexin–dynein regulatory complex (GAS8), radial spokes (RSPH1), or fibrous sheath (AKAP4). (B)Immunostaining of predicted LRGUK partners (CFAP70, MYCBPAP, HOOK1, and HOOK2) also showed no difference between control and patient. (C) IFT20 staining similarly appeared comparable between control and patient spermatozoa, suggesting no overt alteration of intraflagellar transport. Scale bars: 10 μm.

## Data Availability

The data that supports the findings of this study are available in the Supporting Information of this article.
